# The global regulator FpLaeB is required for the regulation of growth, development, and virulence in *Fusarium pseudograminearum*

**DOI:** 10.3389/fpls.2023.1132507

**Published:** 2023-02-22

**Authors:** Yuxing Wu, Yajiao Wang, Sen Han, Qiusheng Li, Lingxiao Kong

**Affiliations:** Institute of Plant Protection, Hebei Academy of Agricultural and Forestry Sciences, Integrated Pest Management Center of Hebei Province, Key Laboratory of IPM on Crops in Northern Region of North China, Ministry of Agriculture, Baoding, China

**Keywords:** *Triticum aestivum*, deletion mutant, conidiation, secondary metabolite, deoxynivalenol

## Abstract

*Fusarium pseudograminearum* is a soil-borne pathogen that is capable of causing a highly destructive crown disease in wheat. Secondary metabolites (SMs), especially deoxynivalenol (DON), are the primary virulence factors during infection. Here, we characterised the global regulator *FpLaeB*, an orthologue of LaeB protein function, to regulate the SM in *Aspergillus nidulans*. Through the utility of the gene targeting approach, we found that the vegetative growth of the *FpLaeB* deletion mutant was drastically reduced compared to that of the wild type. *FpLaeB* was also important for conidiation because the *FpLaeB* deletion mutant formed fewer conidia in induced medium. In addition, the sensitivity of the *FpLaeB* deletion mutant to the cell wall integrity inhibitor was decreased, while its growth was more severely inhibited by the cell membrane inhibitor sodium dodecyl sulfate (SDS) than that of the wild type. More importantly, the virulence was decreased when the *FpLaeB* deletion mutant was inoculated onto the wheat stem base or head. Through genome-wide gene expression profiling, *FpLaeB* was found to regulate several processes related to the above phenotypes such as the carbohydrate metabolic process, which is an integral and intrinsic component of membranes, especially SMs. Furthermore, the generation of DON was impaired in the *FpLaeB* deletion mutant *via* ultraperformance liquid chromatography tandem mass spectrometry (UPLC-MS/MS) assay. These results showed that *FpLaeB* plays an important role in the growth, development, and maintenance of the cell wall, and in membrane integrity. More importantly, *FpLaeB* is required for SMs and full virulence in *F. pseudograminearum*.

## Introduction

The soil-borne pathogen *Fusarium pseudograminearum* is capable of causing *Fusarium* crown rot, a highly destructive worldwide disease resulting in yield losses of up to 10%–35% in a normal year in Australia and the Northwestern United States (Smiley et al., 2005; Murray and Brennan, 2009). In particular, this chronic disease is an increasing concern in the Huanghuai region of China including Henan, Hebei, and Shandong provinces (Li et al., 2012; Ji et al., 2016). The colonisation of *F. pseudograminearum* seems to occur at the coleoptile. Then, the infectious growth spreads to leaf sheaths and subcrown internodes with extensive browning. Severely diseased plants may result in white heads containing either no or shrivelled grains (Kazan and Gardiner, 2018). Similar to other *Fusaria*, some secondary metabolites (SMs) like the trichothecene toxin deoxynivalenol (DON) can contribute to the virulence in *F. pseudograminearum* (Monds et al., 2005; Tunali et al., 2012; Powell et al., 2017). Thus, the analyses of genes or regulation related to SM could reveal potential roles for the development or pathogenic life cycles of *F. pseudograminearum*. Fungal SM biosynthesis has been regulated in a complex process. The different regulations include signal transduction pathways, epigenetic modifications, and pathway-specific and global regulators (Brakhage, 2013). Global regulators including response to ambient light, carbon and nitrogen sources, and pH have been identified in several fungi (Chen et al., 2019). LaeA is a global regulator for sterigmatocystin and penicillin biosynthesis found in *Aspergillus nidulans* (Bok and Keller, 2004; Bayram et al., 2008). In addition, the regulation of secondary metabolism by LaeA has been characterised in other fungi, such as gliotoxin biosynthesis in *A. fumigatus* and lovastatin biosynthesis in *A. terreus* (Brakhage, 2013). In *Fusarium graminearum*, the expression of seven TRI genes was reduced in the FgLaeA deletion mutant. The accumulation of 15A-DON was abolished as well. The deletion of FgLaeA also leads to a 30-fold reduction of Zearalenone (Hee-Kyoung et al., 2013). Recently, a new global regulator, LaeB, involved in regulating sterigmatocystin production similar to LaeA, was identified using a forward genetic screening in *A. nidulans*. The LaeB protein contains a transcription initiation factor IIA (TFIIA) domain and a G-protein pathway suppressor domain. The two domains have low homology (Pfannenstiel et al., 2017). The LaeB deletion mutant exhibited a clear colour change compared to the wild type in *A. nidulans*. The majority of metabolites decreased or disappeared in the LaeB deletion mutant comprising the recipient strain. Meanwhile, some newly produced compounds were detectable (Lin et al., 2018). All these results suggested that most SM gene clusters should be regulated by LaeB in *A. nidulans*.

In light of the regulation effects of LaeB on SMs in *A. nidulans*, the biological functions of the plant pathogenic fungus *F. pseudograminearum* need to be determined to understand the intricate roles of SMs—important virulence factors that are regulated by its homologue—and in which manner this regulation may occur. Functional analysis of LaeB might provide a novel insight to understand the development and pathogenicity of *F. pseudograminearum*.

In this study, the effect of the LaeB orthologous gene *FpLaeB* in vegetative growth, conidiation, virulence, sensibility of abiotic stresses, and expression of SM genes was investigated in *F. pseudograminearum.* In addition to attenuated growth, *FpLaeB* has made a difference in conidiation and maintenance of cell wall and cell membrane integrity. Moreover, the *FpLaeB* gene disruption mutant drastically impaired virulence. *FpLaeB* was found to regulate the expression of genes related to the above phenotype such as the carbohydrate metabolic process and the integral and intrinsic component of membranes, especially SMs. The generation of DON was impaired in the *FpLaeB* deletion mutant as well. These results indicate that *FpLaeB* is involved in the growth, development, virulence, and SMs of *F. pseudograminearum*.

## Materials and methods

### Strains and growth conditions

The wild-type strain of *F. pseudograminearum* named 2035 was preserved by the Laboratory of Fungi Diseases in the Institute of Plant Protection, Hebei Academy of Agricultural and Forestry Sciences, PRC. The wild-type and mutant strains were activated and cultured on potato dextrose agar (PDA, 20% potato extract, 2% dextrose, and 1.5% agar) medium in this study.

The growth rates of different strains were expressed as colony radius per day on PDA medium at 25°C. For the conidiation assay, different strains were grown on carboxymethylcellulose sodium (CMC) medium for 4 days. A hemocytometer was used to determine the concentration of conidia (Chen et al., 2021). To assay stress responses, mutants and wild-type strains were grown on synthetic medium (STM) [0.05% yeast extract, 0.5% (NH_4_)_2_SO_4_, salts (0.15% KH_2_PO_4_, 0.06% CaCl_2_, and 0.06% MgSO_4_), and trace amounts of metals (0.0005% FeSO_4_ 7H_2_O, 0.00016% MnSO_4_ H_2_O, 0.00037% CoCl_2_, and 0.00014% ZnSO_4_ 7H_2_O)] containing NaCl (0.7 M), H_2_O_2_ (3 mM), Congo red (CR; 200 mg/L), or SDS (0.01%). Colony diameter was measured after incubation for 4 days. During gene deletion or complementarity, TB_3_ (0.3% yeast extract, 0.3% casamino acids, 20% sucrose, and 1.5% agar) medium mended with hygromycin B (250 μg/ml, Calbiochem, La Jolla, CA) or geneticin (250 μg/ml, Sigma, St. Louis, MO) has been used to select resistant transformants.

### *FpLaeB* gene deletion and complementarity

The LaeB homology protein *FpLaeB* was identified *via* querying the *F. pseudograminearum* genomic sequence (GenBank accession NC_031951.1). The conserved domains of *FpLaeB* were predicted *via* the Conserved Domain Search Service (CD Search) in the National Center for Biotechnology Information (NCBI). The phylogenetic tree of *FpLaeB* and its homology proteins were constructed *via* the neighbour-joining method with the MEGA version 7.02 software package (Xia et al., 2021).

Open reading frame (ORF) was replaced by the hygromycin phosphotransferase gene to construct the deletion mutant of *FpLaeB*. Two primer pairs FpLaeB-1F/2R and FpLaeB-3F/4R were used to amplify the upstream and downstream flanking fragment of the *FpLaeB* gene. The hygromycin phosphotransferase (*hph*) gene was amplified *via* the primer pair HYG-F/R. The replacement fragment was constructed by joining the three fragments *via* double-joint polymerase chain reaction (PCR) (Yu et al., 2004). The *FpLaeB* replacement fragment was transformed into protoplasts of wild-type 2035 by the polyethylene glycol (PEG) approach (Liu and Friesen, 2012). Following screening by hygromycin, the transformants were screened and confirmed using PCR and Southern blot analyses, respectively (Tang et al., 2018). For complementation assays, XhoI-digested pFL2 and the *FpLaeB* fragments with promoters cotransformed into yeast strain XK1-25. *FpLaeB*–pFL2 plasmid was constructed by the yeast gap repair method (Zhang et al., 2017). Then, *FpLaeB*–pFL2 was transformed into the protoplasts of the *FpLaeB* deletion mutant by the PEG approach as well. After geneticin screening, the primer pair FpLaeB-5F/6R was used to confirm the complementation strain from geneticin-resistant transformants. Primers used for deletion, complementarity, and gene expression are listed in **Supplementary Table S1**.

### Plant infection assays

For virulence on the wheat stem base, conidia were collected from CMC according to the method used in strains and culture conditions and then diluted to a concentration of 10^5^ conidia/ml. The inoculation procedure was as described by Li et al. (2009) with the following modifications: Seeds of susceptible cultivar Shixin 828 were germinated on wet filter paper saturated in Petri dishes. Germinated seeds were immersed in the spore suspension for 1 min. Then, treated seeds were sown in a pot with a diameter of 15 cm containing sterile soil mix. There were three replicates, with each pot containing 20 seedlings. The severity of *Fusarium* crown rot was assessed at 35 days post-inoculation (dpi) using a 0–5 scale (Li et al., 2009).

For virulence on wheat heads, conidia of different strains were diluted to a concentration of 10^5^ conidia/ml in 0.01% (vol/vol) Tween 20. A 20-μl aliquot of conidial suspension was injected into a floret of a wheat head of susceptible cultivar Shixin 828 at early anthesis. There were 30 replicates for each strain. The severity of head blight used a scale of 0–4 (Wang et al., 2015).

The severity of plants was determined using the disease index. The disease index (DI) was calculated as follows: DI = [∑ (number of diseased plants in this scale × value of this scale)/(total number of plants investigated × highest value of scale)] × 100.

### RNA-seq and bioinformatics analysis

Both wild-type and mutant strains were transferred to PDA and incubated at 25°C for 3 days. Mycelia samples of three biological repetitions were collected from the surface of the colony. Total RNA of the wild-type and FpLeaB deletion mutant were extracted using an RNA extraction kit. The process followed the manufacturer’s instructions (Qiagen, Hilden, Germany). Novogene Co., Ltd. (Tianjin, China) conducted the library preparation and sequencing procedure. Clean reads for each sample were mapped on the reference genome of *F. pseudograminearum* CS3096 (Gardiner et al., 2012) using the TopHat 2.0.8 software with default parameters. The mapped read counts were used to determine the number of reads per kilobase per million reads (RPKM). The HTSeq v0.9.1 software was used to identify the different expressions of genes between mutant and wild-type strains (Trapnell et al., 2010). The DESeq2 software was used to isolate the differentially expressed genes (DEGs) with false discovery rate adjusted *p* < 0.05 (Love et al., 2014). The RPKM value of the same gene was used to calculate the fold change (FC) in log2(FC) greater than 1.0 between mutant and wild type. Gene ontology (GO) annotation was implemented by the GOseq package software (Young et al., 2010). The clusterProfiler v3.8.1 software was used to analyse the Kyoto Encyclopedia of Genes and Genomes (KEGG) enrichment with *p* < 0.05.

Quantitative real-time PCR (qRT-PCR) was used to determine the transcript levels of SM genes (Wang et al., 2020). Total RNA was isolated from the mycelium using the TRIzol reagent (Invitrogen, Carlsbad, CA) following the manufacturer’s instructions. First-strand complementary deoxyribo nucleic acid (cDNA) was derived from total RNA using the Fermentas 1st cDNA synthesis kit (Hanover, MD) according to the manufacturer’s instructions. All values were calculated and normalised using the 2^−ΔΔCT^ method and *FpActin* (FPSE_04141) gene, respectively (Livak and Schmittgen, 2001; Xia et al., 2021). Mean and standard deviation of data were collected from three biological replicates. Fisher’s least significant difference (LSD) in the Statistical Package for the Social Sciences (SPSS) was used for statistical analysis (*p* < 0.05). The primers are listed in **Supplementary Table S1**.

### Determination of DON production

Three 6-mm-diameter agar plugs taken from the edge of the colony were inoculated into a 150-ml Erlenmeyer flask containing 30 ml of trichothecene biosynthesis induction (TBI) medium (Gardiner et al., 2009). After cultivating at 180 rpm in a shaker at 28°C for 3 days, the mycelium was collected for expression analysis of *TRI5*. The fermentation broth was filtered with a 0.22-μm aqueous filter at 14 dpi. DON was detected *via* ultraperformance liquid chromatography tandem mass spectrometry (UPLC-MS/MS) (Soleimany et al., 2012).

## Results

### Deletion and complementarity of *FpLaeB*

**Figure 1:**
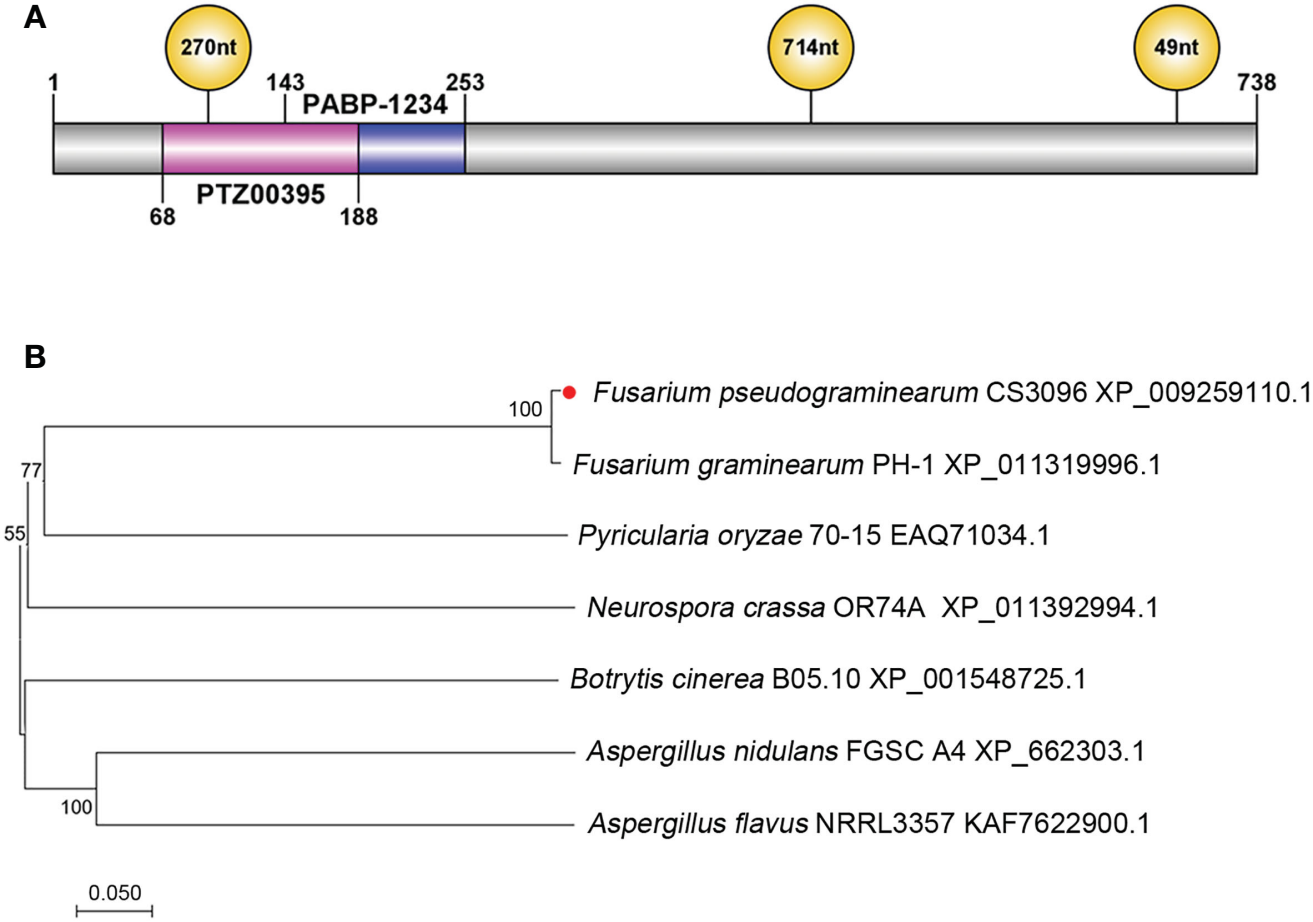
Structures and phylogenetic analysis of *LaeB*/LaeB of *Fusarium pseudograminearum*. **(A)** Location of conserved domains and intron in the FpLaeB protein and gene, respectively. Blue and pink bars represent polyadenylate binding protein domain (PABP-1234) and Sec24-related protein domain (PTZ00395), respectively. Three introns interrupted the *FpLaeB* gene at different positions. **(B)** Phylogenetic analysis of LaeB in *F*. *pseudograminearum* and other fungi. The neighbour-joining method used to analyse the amino acid sequences by the MEGA version 7.02 software package. The numbers at branches represent the supporting percentage of 1,000 bootstrap replicates.

The FpLaeB protein (accession number XP_009259110.1) contains 738 amino acids (aa) and was identified to be 47.99% homologous to *A. nidulans* LaeB (AN4699). A polyadenylate binding protein domain (PABP-1234) and Sec24-related protein domain (PTZ00395) were predicted at 141–251 and 68–188 aa *via* CD Search in NCBI, respectively. The coding gene sequence of FpLaeB was interrupted by three introns at 289–378, 1,487–2,200, and 2,875–2,924 bp (**Figure 1A**). Phylogenetic analysis showed that FpLaeB is a fungal LaeB homologue with a very close genetic relationship to that of *F. graminearum* (**Figure 1B**).

**Figure 2:**
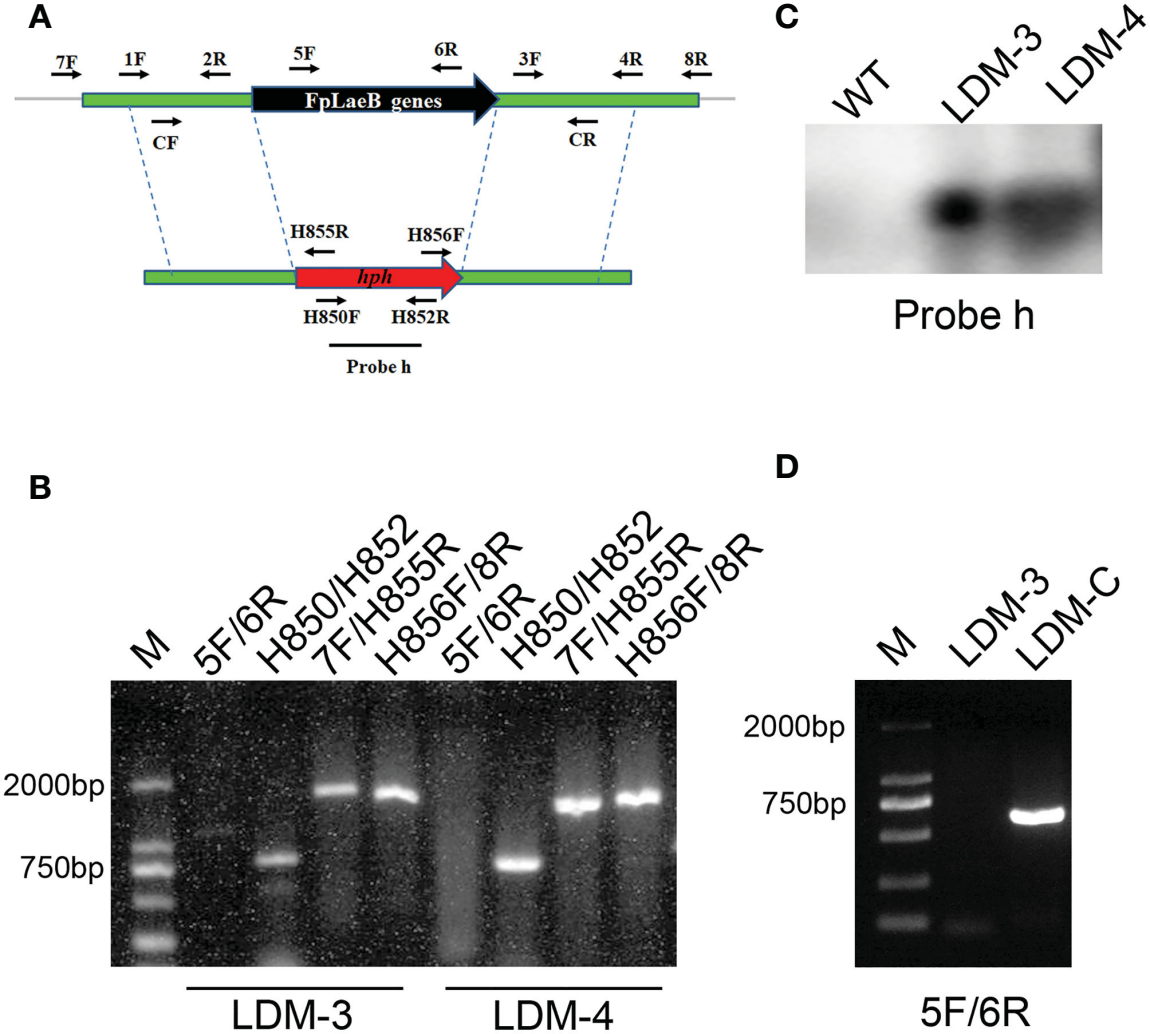
Deletion and complementation of the *FpLaeB* gene in *Fusarium pseudograminearum*. **(A)** The double-joint method was used to generate the *FpLaeB* gene replacement fragment. The arrows mark the positions and directions of primer pairs used for amplifying fragment and detection by PCR. **(B)** The genomic DNA of two deletion mutants was detected using four primer pairs, namely, FpLaeB*-*5F/6R, H850/H852, FpLaeB*-*7F/H855R, and H856F/FpLaeB*-*8R. Four lanes present the target gene, *hph*, and the recombination of upstream and downstream, respectively. **(C)** Genomic DNA of wild-type and two deletion mutants was hybridised using probe h (*hph*) in Southern blots. **(D)** PCR confirmation of complementation using primer FpLaeB*-*5F/6R.

In order to generate the *FpLaeB* deletion mutant (LDM), we used the hygromycin B phosphotransferase (*hph*) gene to replace the entire ORF of *FpLaeB* (**Figure 2A**). The transformants were confirmed by PCR amplification after preliminary screening by hygromycin. There is no PCR product when the primer pairs of ORF (FpLaeB-5F/6R) were used to amplify *FpLaeB* deletion mutants (**Figure 2B**). The genomic DNA of wild-type and mutant strains were further hybridised using the *hph* probe (Probe h). We found that only one 6.3-kb fragment band presented in the *FpLaeB* deletion mutant (**Figure 2C**). Hence, a homologous recombination event occurred in a single locus in the *FpLaeB* deletion mutant. The complementarity strain of *FpLaeB* deletion mutants showed an expected band (LDM-C, **Figure 2D**).

### *FpLaeB* is important for vegetative growth and conidiation

**Figure 3:**
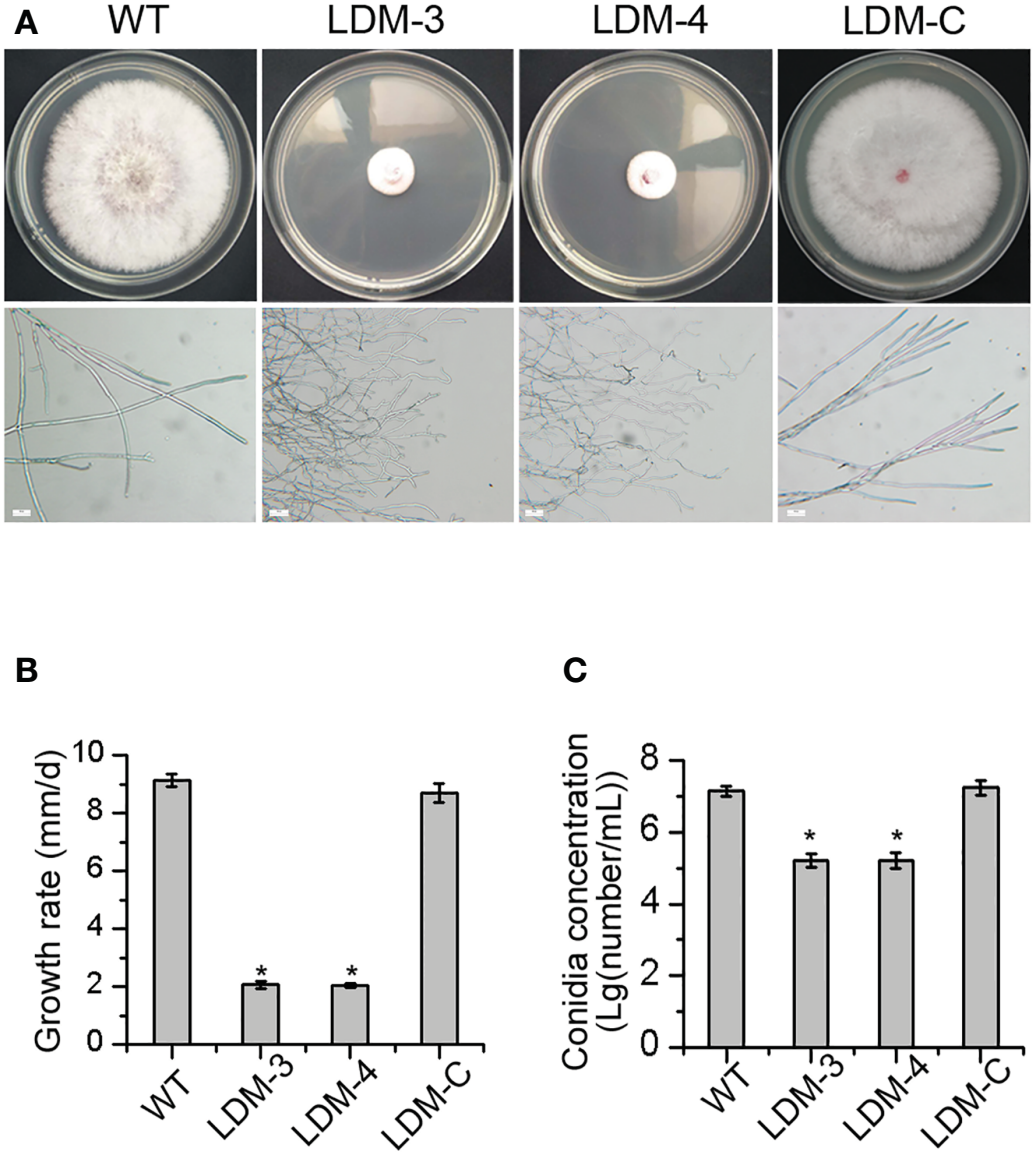
Effects of *FpLaeB* deletion on the morphology and conidiation of *F pseudograminearum*. **(A)** Colonies of wild-type and *FpLaeB* deletion mutant strains on potato dextrose agar (PDA) at 3 days post-inoculation. The photos of hyphal morphology were taken under a 200 times microscope. Bar = 20 μm. **(B)** Growth rate was presented by growth radius per day, which calculated radial growth between 2 and 3 dpi. **(C)** The logarithm of number of conidia per millilitre after 4 dpi in induced medium. Mean and standard deviation of data were calculated from three biological replicates. Statistical analysis was performed using Fisher’s least significant difference (LSD) in the Statistical Package for the Social Sciences (SPSS). Asterisks represent a significant difference between mutants and wild type (*p* < 0.05).

To evaluate whether *FpLaeB* was involved in the morphology formation of *F. pseudograminearum*, the deletion mutant of *FpLaeB* and wild-type strains were cultivated on PDA medium. The colonial morphology of *FpLaeB* deletion mutant strains was dramatically affected compared to wild type. There is scarcely any aerial hyphal growth in deletion mutants in contrast to the wild type. The colonies displayed a more compact appearance and a shorter peripheral edge in deletion mutants. Also, the hyphae are tenuous and curved under the microscope (**Figure 3A**). The hyphal growth rate of the *FpLaeB* deletion mutant was quantified to be 23% that of the wild type (**Figure 3B**). When the *FpLaeB* genes were reintroduced into the deletion mutant strain, the morphology formation of the complemented mutant was restored to that of the wild type (**Figures 3A, B**). Thus, the *FpLaeB* showed an important role on the growth phenotype of *F. pseudograminearum*.

To test whether *FpLaeB* affected conidiation, we assayed the conidia concentration of different strains in induced media. After 4 dpi, the number of conidia was 10^7^/ml in the wild-type strain. However, this amount was 10^5^/ml in the deletion mutant at the same time. When reintroducing the *FpLaeB* genes into deletion mutant strains, the phenomenon could be reversed. This result showed the regulated effect of *FpLaeB* on conidiation in *F. pseudograminearum* (**Figure 3C**).

### *FpLaeB* affects sensibility to cell membrane and the cell wall integrity inhibitor

**Figure 4:**
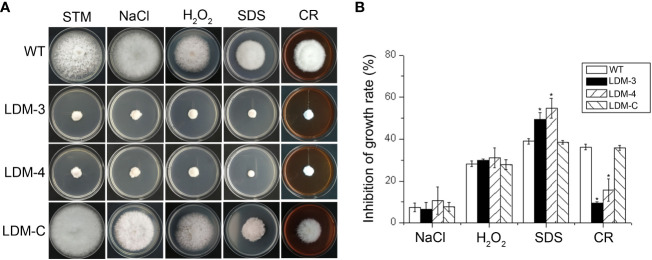
Effects of *FpLaeB* deletion on sensibility to abiotic stress in *F pseudograminearum*. **(A)** The wild-type, *FpLaeB* deletion mutant, and complemented mutant strains grew on synthetic medium (STM) mended with NaCl, H_2_O_2_, SDS, or Congo red (CR). Pictures were taken after 3 dpi on stress media. **(B)** Inhibition rates were calculated by the growth rate of stress media compared with that of the STM without inhibitor. Mean and standard deviation of data were calculated from three biological replicates. Statistical analysis was performed using Fisher’s LSD in SPSS. Asterisks on the bars represent statistically significant difference compared to wild type (*p* < 0.05).

To characterise whether *FpLaeB* affects sensibility to abiotic stress, the deletion mutants and wild-type strains were inoculated on STM mended with 3 mM H_2_O_2_ (oxidative stress), 0.7 M NaCl (Na^+^, osmotic pressure), 0.01% SDS (cell membrane damaging agent), or 200 mg/L CR (cell wall inhibitor, **Figure 4A**). We assayed the inhibition rates of *FpLaeB* deletion mutants, which were higher than those of wild type on STM mended with 0.01% SDS. The inhibition rates of *FpLaeB* deletion mutants were lower than those of the wild-type strain on STM mended with 200 mg/L CR. There are no significant differences in sensibility between deletion mutants and the wild-type strain when cultured on NaCl and H_2_O_2_ media (**Figure 4B**). The phenomenon was restored when the *FpLaeB* genes were reintroduced into the deletion mutant strain. These results indicated that FpLaeB is important to maintain cell wall and membrane integrity in *F. pseudograminearum*.

### *FpLaeB* is required for plant infection

**Figure 5:**
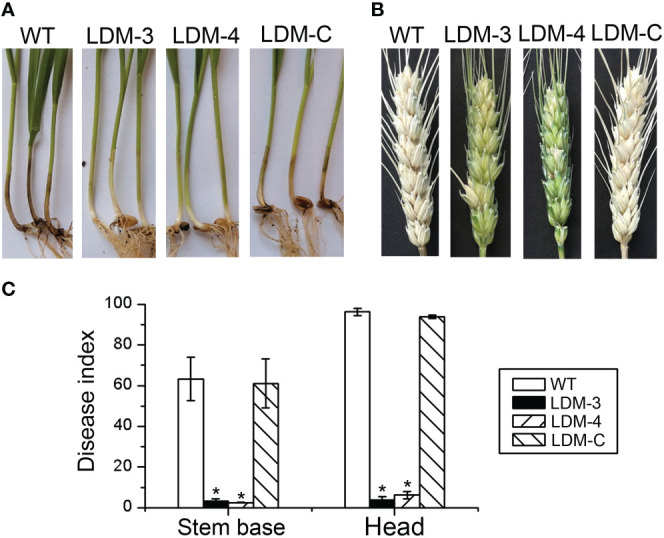
Effects of *FpLaeB* deletion in plant infection. **(A)** Images were taken when wild-type, *FpLaeB* deletion mutant, and complemented mutant strains were inoculated to the stem base during the seedling stage at 21 days. **(B)** Flowering wheat heads inoculated with the different strains were photographed at 21 dpi. **(C)** Virulence is represented by the disease index. The disease index is calculated from plants with different disease grades. The experiments were repeated three times. Statistical analysis was performed by using Fisher’s LSD in SPSS. Asterisks on the bars represent statistically significant difference compared to wild type (*p* < 0.05).

We assay pathogenicity tests with stem base and flowering wheat heads to characterise the role of *FpLaeB* in disease development. The wild-type strain developed typical crown rot and head blight symptoms at the stem base and heads at 21 dpi, respectively. Under the same condition, limited discolouration appeared only at the inoculation site of the *FpLaeB* deletion mutant (**Figure 5A**). The disease index of the *FpLaeB* deletion mutant was reduced by approximately 95% in the crown and head compared to that of wild type. To confirm these findings, we performed a complementation assay with the *FpLaeB* gene. The complementation led to a normal phenotype in both cases (**Figure 5B, 5C**). This result confirmed that *FpLaeB* is involved in virulence in *F. pseudograminearum*.

### *FpLaeB* regulates gene expression including membrane and secondary metabolism

**Figure 6:**
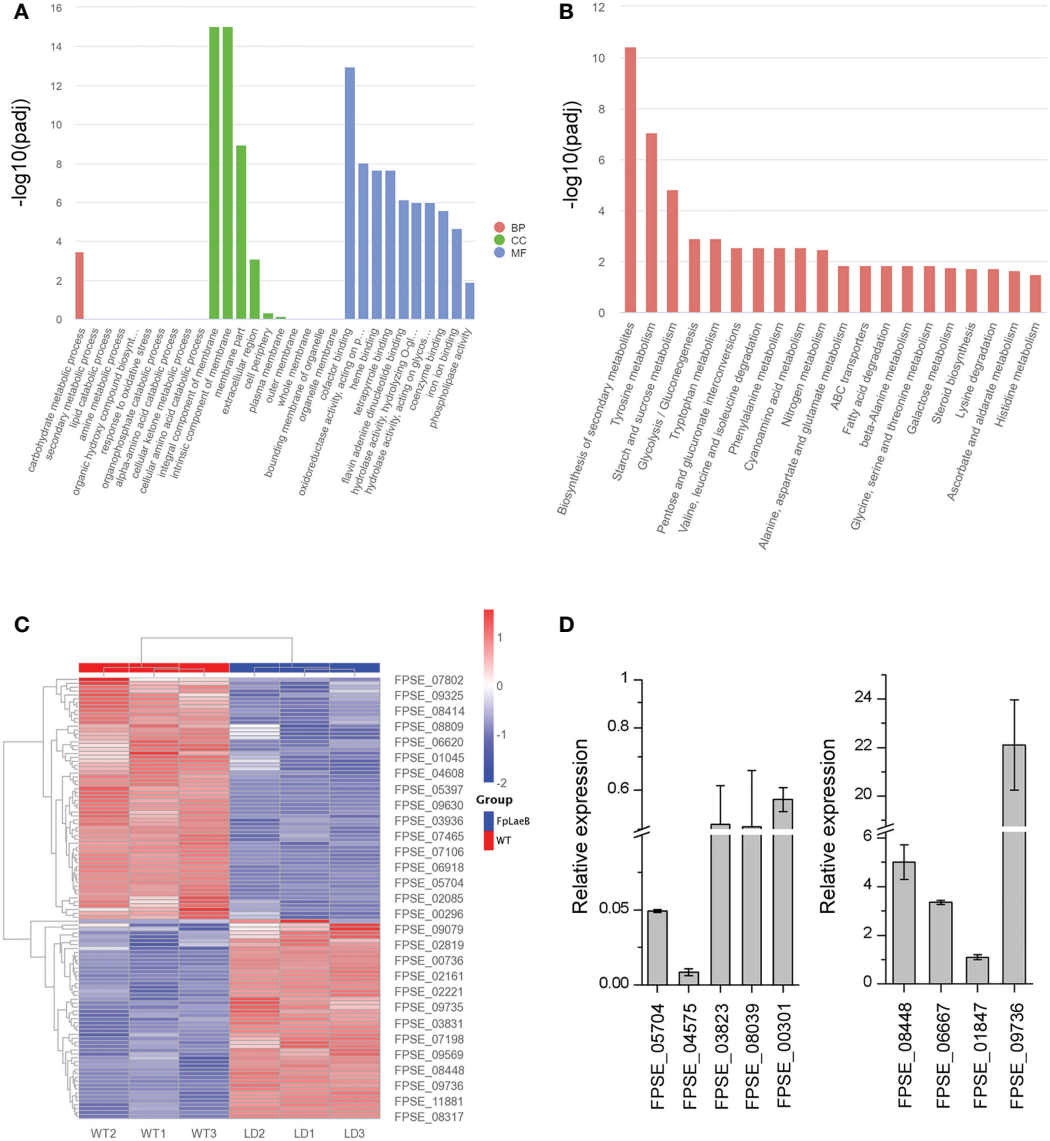
Transcriptome analysis of differentially expressed genes (DEGs) between the *FpLaeB* deletion mutant and wild type. **(A)** Bar plot of Gene ontology (GO) annotation of DEGs. The DEGs were categorised into three main categories: biological process (BP), cellular component (CC), and molecular function (MF). The DEGs were log2(FC) greater than 1.0 with a threshold at *p* and corrected *p* < 0.05. **(B)** Kyoto Encyclopedia of Genes and Genomes (KEGG) pathway enrichment analysis of DEGs. **(C)** Heatmap for differentially regulated genes encoding secondary metabolites (SMs). The different colour scale presents the counts of expression normalised by *Z*-score. **(D)** Real-time qPCR for nine DEGs encoding SM including five upregulated genes and four downregulated genes between wild-type and *FpLaeb* mutants. Cycle threshold of *FpActin* gene was used to normalise the two samples. Expression levels of these genes in wild type were arbitrarily given a value of 1. Mean and standard deviation of data were calculated from three biological replicates. Statistical analysis was performed using Fisher’s LSD in SPSS.

We analysed transcriptomes (RNA-seq) from wild type and the *FpLaeB* deletion mutant [raw sequence data for RNA-seq data are available in the NCBI Sequence Read Archive (SRA), accession number: PRJNA914495] to reveal the regulatory role of *FpLaeB* at a genome-wide scale. The absence of *FpLaeB* caused a significant change in expression levels in more than 3,200 genes in the *F. pseudograminearum* genome. The number of differentially downregulated genes was 1,748, while 1,456 genes showed an increase in their expression [all the expression analysis at *p* < 0.05, log2(FC) >1 or <−1] (**Supplementary Table S2**). Three classes, namely, “molecular function”, “cellular components”, and “biological process”, of the gene product were used to define the GO. In the class cellular components, the two most populated categories were “integral component of membrane” and “intrinsic component of membrane”. This result showed that the majority of both upregulated and downregulated DEGs were significantly associated with “membrane” (**Figure 6A**; **Supplementary Table S3**). We observed a broad range of transcripts encoding the biosynthesis of SMs that were enriched during Kyoto Encyclopedia of Genes and Genomes (KEGG) pathway analysis (**Figure 6B**; **Supplementary Table S4**). There were 62 gene-encoded SMs with significantly downregulated expression while 53 genes were upregulated in *FpLaeB* (**Figure 6C**). To validate the expression profiles of these SMs, the expression levels of nine genes encoding SMs were used to verify the accuracy of transcriptomes. The results show that the expression levels (five downregulation and four upregulation) were basically the same between transcriptomes and qRT-PCR (**Figure 6D**).

### *FpLaeB* is important for DON production

**Figure 7:**
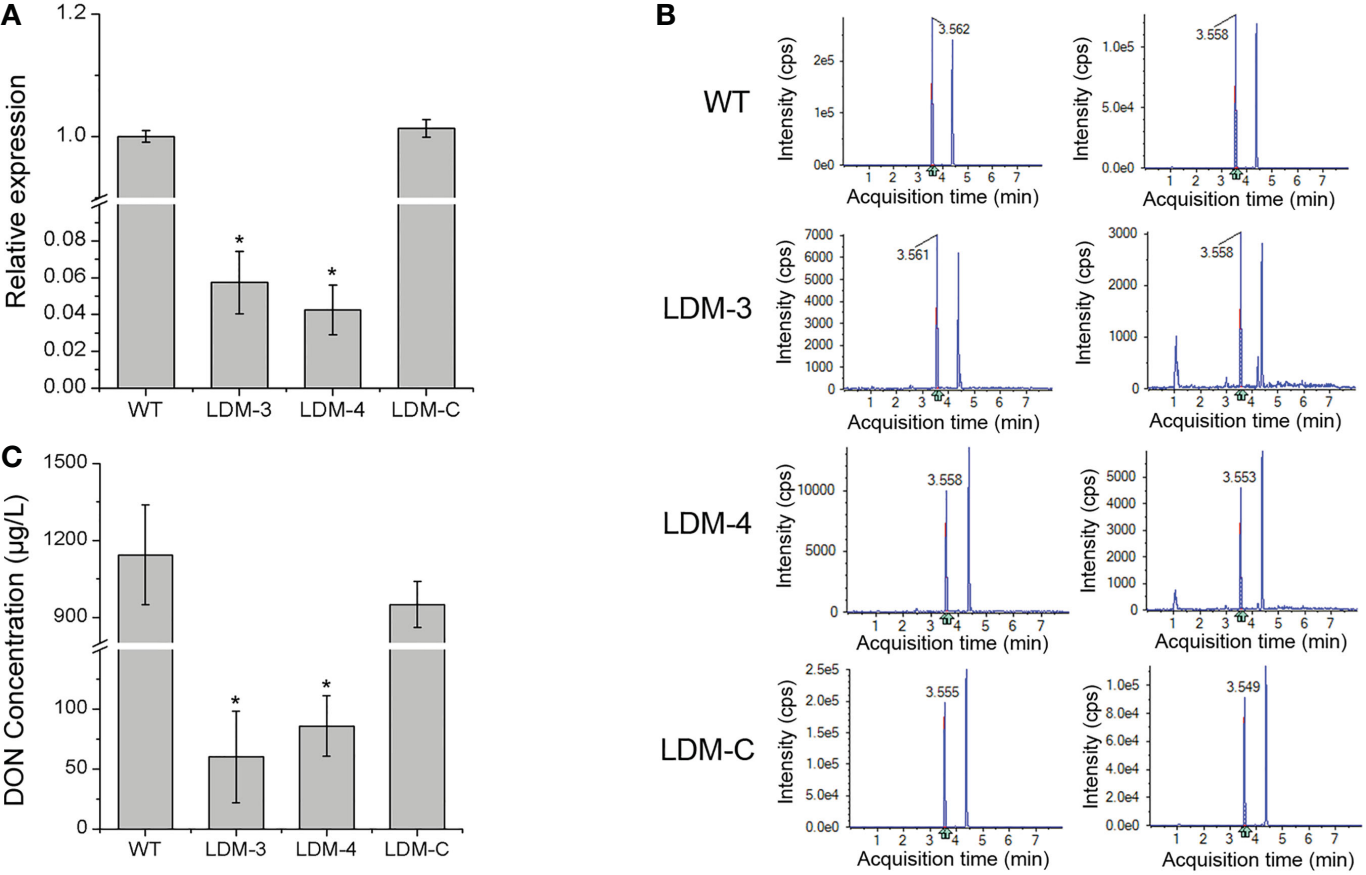
Transcription level of the TRI5 gene and DON concentration in inducing medium. **(A)** Relative transcript abundances of the TRI5 gene in mycelium in inducing medium were compared between the wild-type and *FpLaeB* mutant strain at 7 dpi. Cycle threshold of the FpActin gene was used to normalise different samples. Expression levels of wild type were arbitrarily given a value of 1. **(B)** UPLC-MS/MS chromatogram of DON at inducing media of different strains. **(C)** DON concentration of different strains cultured in inducing medium for 14 days. Mean and standard deviation of data were calculated from three biological replicates. Statistical analysis was performed using Fisher’s LSD in SPSS.

Furthermore, we monitored the transcription level of the *TRI*5 gene and the production of DON in the *FpLaeB* deletion mutant and the wild-type strain in inducing medium. In comparison with the wild type, the expression levels of the *TRI*5 gene were downregulated 20 times in the *FpLaeB* deletion mutant (**Figure 7A**). After induced culture, the DON concentration of the culture solution in the deletion mutant strain was 60.34 µg/L. This value was significantly lower than that of the wild-type strain, which was 1,143.83 µg/L (**Figures 7B, C**). Therefore, *FpLaeB* appears to play an important role in regulating the *TRI* gene expression and DON production of *F. pseudograminearum*.

## Discussion

The LaeB protein is identified as a novel transcriptional regulator of the sterigmatocystin in *A. nidulans*. It is required for the transcription of *aflR*, the transcriptional regulator of the sterigmatocystin (Woloshuk et al., 1994; Pfannenstiel et al., 2017). In order to explore the regulating function of LaeB in the secondary metabolism (SM) of *F. pseudograminearum*, we set out to assess the importance of a putative LaeB homologue, FpLaeB, based on its homology to *A. nidulans*. This study found that the deletion of *FpLaeB* has drastically impacted the growth rate of mycelia in *F. pseudograminearum*. This result differs from that of a previous study in which the deletion of *LaeB* did not impact the growth rate of *A. nidulans* (Lin et al., 2018). Another homologous protein of LaeB named RsdA has been identified through the genome-wide deletion of regulators in the endophytic fungus *Pestalotiopsis fici*. Deletion of *RsdA* resulted in moderate growth reduction (Zhou et al., 2019). This finding suggests that the function of LaeB could be species-specific in fungus. The roles of LaeB could be divergent in different fungi as well.

In addition to affecting growth rate, FpLaeB also affected the conidiation, sensitivity to SDS and CR stress, and virulence in *F. pseudograminearum*. The regulation of virulence by LaeB is of great significance in the study of pathogenic fungi. In the present study, we demonstrated that FpLaeB plays crucial roles in virulence. Because the deletion mutant impaired growth and development, the reduction in virulence was partially due to the growth defect of the deletion mutant. However, the reduction of the disease index in the stem base and head (approximately 95%) is not proportional to the 77% reduction in growth rate on PDA. Therefore, other reasons should contribute to the reduction of virulence. As a global regulator, the regulatory role of LaeB is extensive in fungi. Other global regulators such as LaeA and velvet complex proteins played crucial roles in the regulation of morphology, development, SM, and virulence in several fungi (Bok et al., 2005; Sarikaya-Bayram et al., 2015; Wang et al., 2019; Maor et al., 2021). They are involved in the regulation of different metabolic pathways, the most significant of which was the effect on secondary metabolism. For example, the expression of at least 9.5% of genes of the *A. fumigatus* genome was regulated by LaeA, wherein the positive control SM biosynthesis genes such as polyketide synthases, P450, nonribosomal peptide synthetases (NRPSs), and monooxygenases amounted to 20% to 40% (Perrin et al., 2007). Also, transcriptomic and proteomic analyses indicated that *VmLaeA* performs both SM transport and biosynthesis in *Valsa mali* (Feng et al., 2020). In addition, FgVeA is involved in various cellular processes including soluble N-ethylmaleimide – sensitive factor attachment protein receptors (SNARE) interactions in vesicular transport and peroxisome biogenesis pathway in *F. graminearum* (Jiang et al., 2011). In the present study, the deletion of *FpLaeB* can affect a quarter of the genes in the *F. pseudograminearum* genome. This result coincided with the severe impact of *FpLaeB* in multiple developmental processes. The highlighted DEGs of the biological process involved the carbohydrate metabolic process according to the GO enrichment statistics (**Supplementary Table S3**). In *Penicillium expansum*, the disturbing carbohydrate metabolic process could lead to growth defect (Lai et al., 2021). Hence, the regulation of the carbohydrate metabolic process by *FpLaeB* is perhaps associated with growth rate in *F. pseudograminearum*. The most severe DEGs of the *FpLaeB* mutant were “membrane” genes. The most DEGs regulated by *FpLaeB* were membrane protein-coding genes, including “intrinsic component of membrane”, “integral component of membrane”, and “membrane part”. This finding seemed to be consistent with the increased sensitivity to SDS of mutants.

*LaeB* is indispensable for the biosynthesis of aflatoxin in *A. flavus* and sterigmatocystin in *A. nidulans* (Pfannenstiel et al., 2017). On the other hand, some novel polyketides have been discovered by the deletion of *LaeB* in *A. nidulans* (Lin et al., 2018). Although this regulator is conserved in *Aspergilli*, the function is not sterigmatocystin specific. In *P. fici*, the deletion of *rsdA* significantly reduces SMs such as asperpentyn, ficiolide A, and chloroisosulochrin. In contrast, in the *rsdA* deletion mutant, six known compounds were isolated, including a new non-ribosomal peptide that was isolated from *P. fici* for the first time. In addition, melanin was significantly accumulated in the mycelium. All these results showed that the deletion of *rsdA* results in significant differences in SM (Zhou et al., 2019). The regulation of SM by *FpLaeB* was proven again in this study. Not only did the highest expression levels and the most differential genes involve biosynthesis of the SM pathway, but also the decrement of pathogenicity-related DON was further clarified in the *FpLaeB* deletion mutant. DON was positively regulated by *FpLaeB*, and this regulation seemed to be involved in virulence in *F. pseudograminearum.* The model of RsdA regulation of fungal SM was proposed based on the genome-wide expression profile and with reference to the SM regulatory network in *P. fici*. It is proposed as an upstream regulator of velvet complex proteins for the regulation of SM, although velvet complex proteins were confirmed to be involved in the regulation of morphology, development, SM, and virulence in both *F. graminearum* and *F. pseudograminearum* (Jiang et al., 2011; Merhej et al., 2012; Gardiner et al., 2021). More research is needed to confirm this model in *F. pseudograminearum.*

In conclusion, we have identified that FpLaeB is important for the vegetative growth, conidiation, sensibility of cell wall and membrane inhibitors, and virulence in *F. pseudograminearum*. Also, a lot of downstream genes have been detected by genome-wide associations of gene expression analysis. In particular, the positive regulation of SM including DON could be related to virulence. Future research should focus on the regulation model of FpLaeB for SM.

## Data availability statement

The datasets presented in this study can be found in online repositories. The names of the repository/repositories and accession number(s) can be at NCBI BioProject accession number: PRJNA914495.

## Author contributions

YXW and LK designed the experiments and managed the projects. YXW, YJW, SH, and QL performed the experiments. YXW, YJW, SH performed the data analysis. YXW wrote the first draft of the manuscript, and LK edited and revised the manuscript. All authors contributed to the article and approved the submitted version.

## Supplementary material

Supplementary Table 1 Primers used in this study.

Supplementary Table 2 All differentially expressed genes between FpLeaB deletion mutant and wild type.

Supplementary Table 3 The main categories of differentially expressed genes on the GO annotations.

Supplementary Table 4 The enriched pathway of differentially expressed genes on the KEGG annotations.

Supplementary Figure 1 Standard curve used to calculate DON content. Regression equation: *y* = 475.79926*x* + 158.33036 (*r* = 0.99925, *r*² = 0.99850).
