# Supplementary figures and images for: The global regulator FpLaeB is required for the regulation of growth, development, and virulence in Fusarium pseudograminearum
Source: Front Plant Sci. 2023 Feb 22;14:1132507. doi: 10.3389/fpls.2023.1132507 (PMC9994621; doi:10.3389/fpls.2023.1132507)

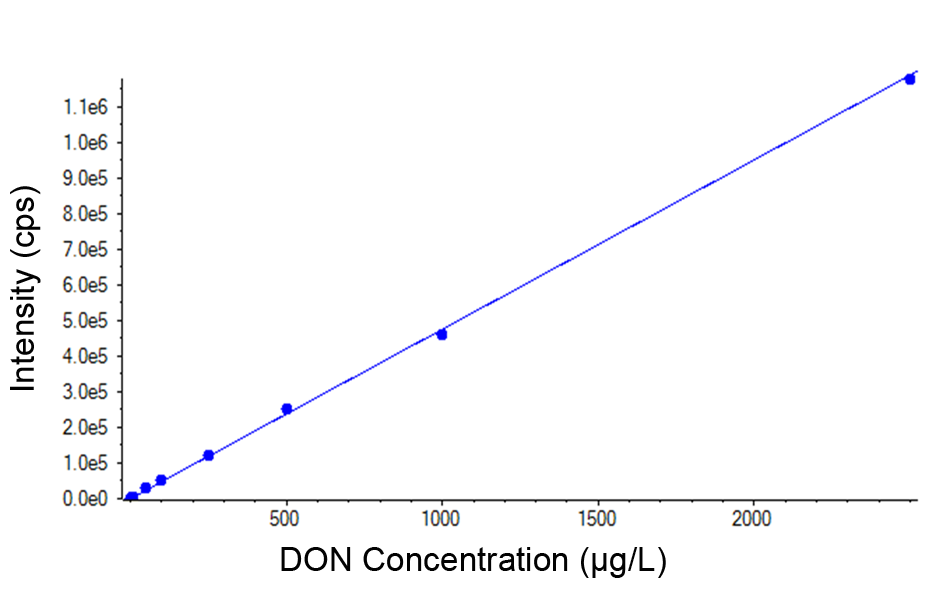

Supplement: Supplementary Figure 1 — Standard curve used to calculate DON content. Regression equation: y = 475.79926x + 158.33036 (r = 0.99925, r² = 0.99850). [file Image_1.tif]
